# p53-Sensitive Epileptic Behavior and Inflammation in *Ft1* Hypomorphic Mice

**DOI:** 10.3389/fgene.2018.00581

**Published:** 2018-11-27

**Authors:** Romina Burla, Mattia La Torre, Giorgia Zanetti, Alex Bastianelli, Chiara Merigliano, Simona Del Giudice, Alessandro Vercelli, Ferdinando Di Cunto, Marina Boido, Fiammetta Vernì, Isabella Saggio

**Affiliations:** ^1^Department of Biology and Biotechnology, Sapienza University of Rome, Rome, Italy; ^2^Nanyang Technological University, Singapore, Singapore; ^3^Neuroscience Institute Cavalieri Ottolenghi, Torino, Italy; ^4^Department of Neuroscience, University of Torino, Piedmont, Italy

**Keywords:** aging, epilepsy, DNA damage, p53, DNA repair

## Abstract

Epilepsy is a complex clinical condition characterized by repeated spontaneous seizures. Seizures have been linked to multiple drivers including DNA damage accumulation. Investigation of epilepsy physiopathology in humans imposes ethical and practical limitations, for this reason model systems are mostly preferred. Among animal models, mouse mutants are particularly valuable since they allow conjoint behavioral, organismal, and genetic analyses. Along with this, since aging has been associated with higher frequency of seizures, prematurely aging mice, simulating human progeroid diseases, offer a further useful modeling element as they recapitulate aging over a short time-window. Here we report on a mouse mutant with progeroid traits that displays repeated spontaneous seizures. Mutant mice were produced by reducing the expression of the gene *Ft1* (*AKTIP* in humans). *In vitro*, AKTIP/Ft1 depletion causes telomere aberrations, DNA damage, and cell senescence. AKTIP/Ft1 interacts with lamins, which control nuclear architecture and DNA function. Premature aging defects of *Ft1* mutant mice include skeletal alterations and lipodystrophy. The epileptic behavior of *Ft1* mutant animals was age and sex linked. Seizures were observed in 18 mutant mice (23.6% of aged ≥ 21 weeks), at an average frequency of 2.33 events/mouse. Time distribution of seizures indicated non-random enrichment of seizures over the follow-up period, with 75% of seizures happening in consecutive weeks. The analysis of epileptic brains did not reveal overt brain morphological alterations or severe neurodegeneration, however, Ft1 reduction induced expression of the inflammatory markers IL-6 and TGF-β. Importantly, *Ft1* mutant mice with concomitant genetic reduction of the guardian of the genome, p53, showed no seizures or inflammatory marker activation, implicating the DNA damage response into these phenotypes. This work adds insights into the connection among DNA damage, brain function, and aging. In addition, it further underscores the importance of model organisms for studying specific phenotypes, along with permitting the analysis of genetic interactions at the organismal level.

## Introduction

Epilepsy comprises a family of disorders characterized by enduring predisposition to generate spontaneous seizures (Scharfman, 2007; Fisher et al., 2014). Seizures are underpinned by multiple mechanisms and their clinical outcome varies widely (Scharfman, 2007). Regardless of their outcome, seizures arise from disruption of mechanisms that create a balance between neuronal excitation and inhibition. Factors corrupting this balance result from alterations at different levels of brain function, from ion channels to receptors, and neuronal circuits (Stafstrom and Carmant, 2015). Epilepsies are often associated with morphological brain abnormalities (Bertram, 2013), but at least one-third have non-structural etiologies (Guerrini et al., 2014). In the last years, concomitantly with human population demographic changes, high incidence of epileptic disorders has been associated with aging, whose specific pathophysiology is under investigation (Leppik and Birnbaum, 2010).

Understanding the mechanistic path to disease is complex in humans due to ethical issues, unavailability of controls and high costs of human research. As a result, studies mostly rely on the use of models, including human 3D cultures and stem cell based systems (Riminucci et al., 2006; Simão et al., 2015), or, for organismal analyses, genetically engineered mice (Baraban, 2007; Saggio et al., 2014; Remoli et al., 2015; La Torre et al., 2018). One of the way in which epilepsy has been modeled in mice is via the inactivation of genes implicated in ion channels (Yu et al., 2006; Baraban, 2007; Glasscock et al., 2012), or of genes encoding for neurotransmitter receptors (Fonck et al., 2005). In addition, epileptic phenomena have been observed in mouse models of Alzheimer disease (Vogt et al., 2011; Ziyatdinova et al., 2011). However, no other genetic models of age related epilepsy have been yet described. Prematurely aging mouse mutants, which recapitulate aging traits over a short time-window (Blasco, 2005; Stewart et al., 2007), offer a specific advantage to model diseases caused or exacerbated by aging, including brain pathologies.

An emerging cause for brain disease and for the aging brain is DNA damage (Langie et al., 2017). DNA integrity poses a challenge for the nervous system as its development depends on a complex series of dynamic and adaptive processes associated to DNA damage (Mckinnon, 2013). Unrepaired DNA lesions have detrimental effects on the developing of a functional nervous system and neural progenitor cells rely on DNA repair systems during the developmental program. After completion of neurogenesis, DNA repair is still of paramount importance to safeguard the genome (Madabhushi et al., 2014), especially to protect the neurons against reactive oxygen species (Langie et al., 2017). DNA damage is also direct cause for cell senescence and for a related inflammatory response (Campisi, 2013; López-Otín et al., 2013).

Mouse mutants of DNA damage functions have opened the path to establish a link between DNA damage and the seizure phenotype (Shen et al., 2010; Bianchi et al., 2017). For example, the inactivation of XRCC1, a central factor in the DNA single strand break repair pathway, leads to profound neuropathology involving behavioral phenotypes consistent with epilepsy (Lee et al., 2009). Data based on studies in *Drosophila* suggest that nuclear architecture and lamins could play a role into epilepsy (Frost, 2016). However, the hierarchy and range of events connecting nuclear architecture, molecular DNA function to epileptic behavior is still to be dissected, along with the elements exacerbating this pathology in aging.

DNA damage affects genome near to randomly, but some chromosomal regions, such as telomeres, are more prone to DNA instability. In mammals, telomeric DNA is composed of double-stranded short tandem repeats of TTAGGG sequence forming higher-order DNA structures binding a specialized protein complex, known as shelterin (de Lange, 2005). We identified a telomere-associated protein named AKTIP in humans (and Ft1 in mouse), which interacts with the shelterin members TRF1 and TRF2 (Burla et al., 2015). AKTIP/Ft1 reduction causes telomere aberrations, DNA instability and cell senescence (Burla et al., 2015). *In vivo*, the genetic reduction of *Ft1* causes premature aging defects including skeletal alterations and lipodystrophy (La Torre et al., 2018). AKTIP/Ft1 interacts with lamins (Burla et al., 2016a,b), pivotal elements for the control of nuclear architecture and DNA function, including DNA repair, replication and transcription (Dittmer and Misteli, 2011). Importantly, *Ft1* mutant mice share similarities with lamin mutant animals, which are models of choice for human progeroid syndromes, linking the *Ft1* model to premature aging and progeroid diseases (Burla et al., 2016a,b, 2018).

Here we report that *Ft1* mutant mice are subjected to repeated seizures. We show that this trait is not linked to overt brain morphological alterations, but is age and inflammation linked. We also demonstrate that this phenotype is sensitive to the expression of the guardian of the genome *p53*, pointing to a role of DNA function in the seizure phenotype.

## Results

### Seizures in Ft1 mutant mice

Mice with reduced levels of *Ft1* were generated using the knock out first (kof) strategy based on the insertion into the target gene (referred as kof allele) of the βgeok cassette (Testa et al., 2004), which traps and truncates *Ft1* nascent transcript reducing the expression of the targeted gene (La Torre et al., 2018). Previous observations of *Ft1*^*kof*/*kof*^ mice revealed that mutant animals display significant reduction in body weight and lifespan, compared to controls. Twenty-one percent of the animals show a severe body size reduction and early post-natal death (we refer to these mice as severely affected *Ft1*^*kof*/*kof*^ mice, abbreviated with SA *Ft1*^*kof*/*kof*^ or SA mutant mice). The leftovers, non SA *Ft1*^*kof*/*kof*^ mice have a mild phenotype, with a median survival of 113 weeks, allowing adult phenotype observation (La Torre et al., 2018).

**Figure 1:**
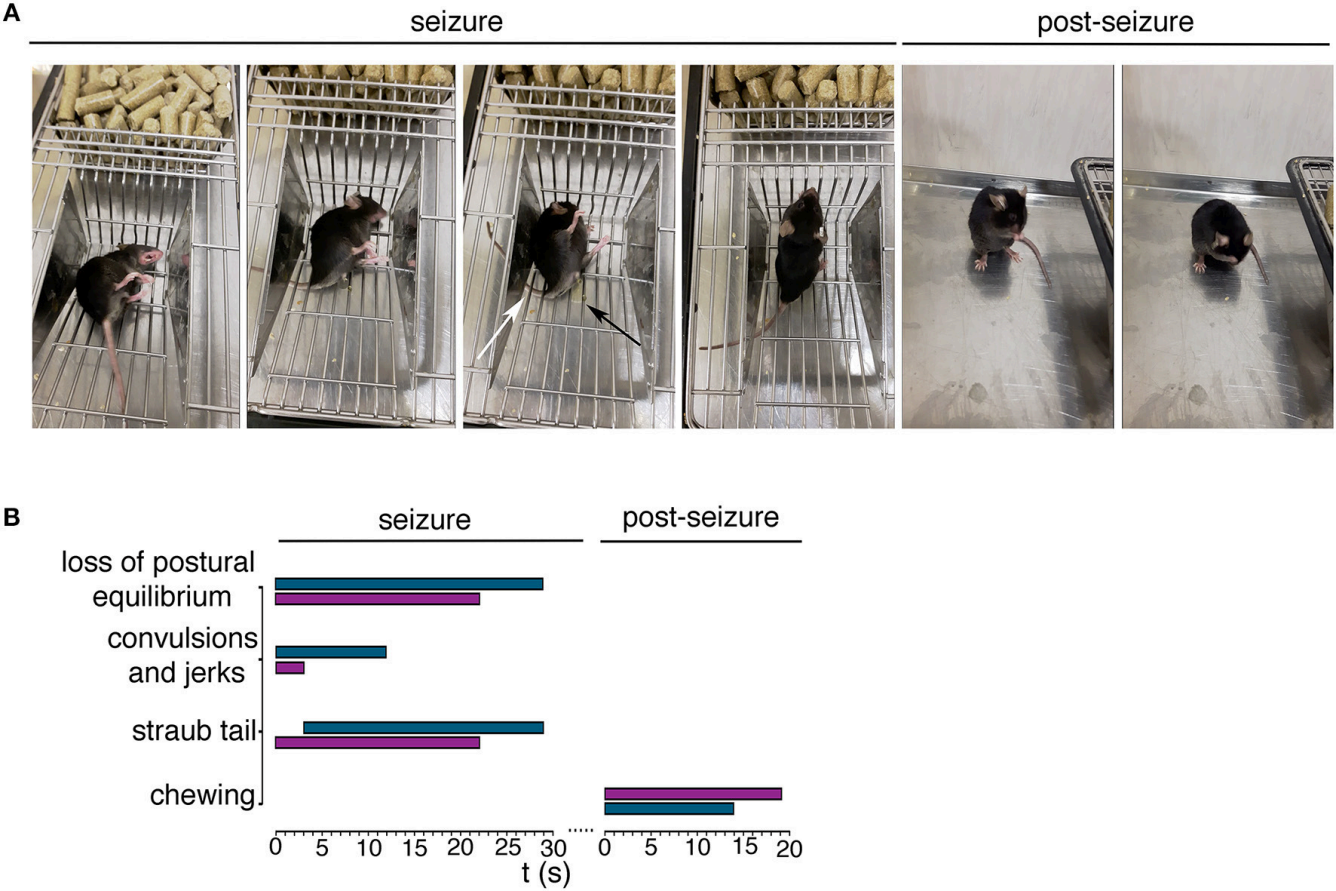
*Ft1*^*kof*/*kof*^ mice exhibit seizures. **(A)** Representative movie frames from non SA *Ft1*^*kof*/*kof*^ mice recording during and post seizure. Starting from left of the seizure panel group: loss of postural equilibrium, arching of the back, Straub tail (third picture, white arrow) and incontinence (third picture, black arrow), recovery of the postural equilibrium. Frames from the movie also show motor automatisms in the post seizure panel group, as chewing (first picture) and grooming (second picture). **(B)** Progression of behavior of two *Ft1*^*kof*/*kof*^ mice, during and post seizure events, each represented by a horizontal bar. The length of each bar indicates the duration of the relative motor type. In turquoise seizure analysis of mouse ID #13489, and in purple the same analysis of mouse ID #16247.

To investigate mutant mouse behavior, we worked on a cohort of animals aged ≥ 21 weeks non SA *Ft1*^*kof*/*kof*^. We recorded spontaneous behavioral abnormalities in non SA *Ft1*^*kof*/*kof*^ mice including episodes of motor tremors and convulsions, fast runs, jumps, and excessive salivation (Figure 1 and Supplementary Movies 1–3). Dissection of video recordings indicated that non SA *Ft1*^*kof*/*kof*^ displayed sudden movements followed by facial twitching, violent hind limbs shaking and falling, back arching, short jerks in muscles of the hind limbs and forelimbs extension, Straub tail and incontinence, followed by short jerks and fast breathing (Figure 1A and Supplementary Movies 1–3). Eventually, animals stood up and returned to normal activity; after few minutes switched to post-ictal phase characterized by short periods of complete immobility, interrupted by short intervals of movement (Supplementary Movies 4–6). Recording showed moving rhythmical up-down or left-right, stroking mouth with forepaws, in a repetitive motion, and appearing to be chewing and grooming (Figure 1A and Supplementary Movies 4–6). Temporal evaluation of the data indicated that non SA *Ft1*^*kof*/*kof*^ mice display a behavioral repertoire corresponding in quality and duration to epileptic phenotypes (Figure 1B), as described for other mouse models (Chabrol et al., 2010; Robie et al., 2017).

**Figure 2:**
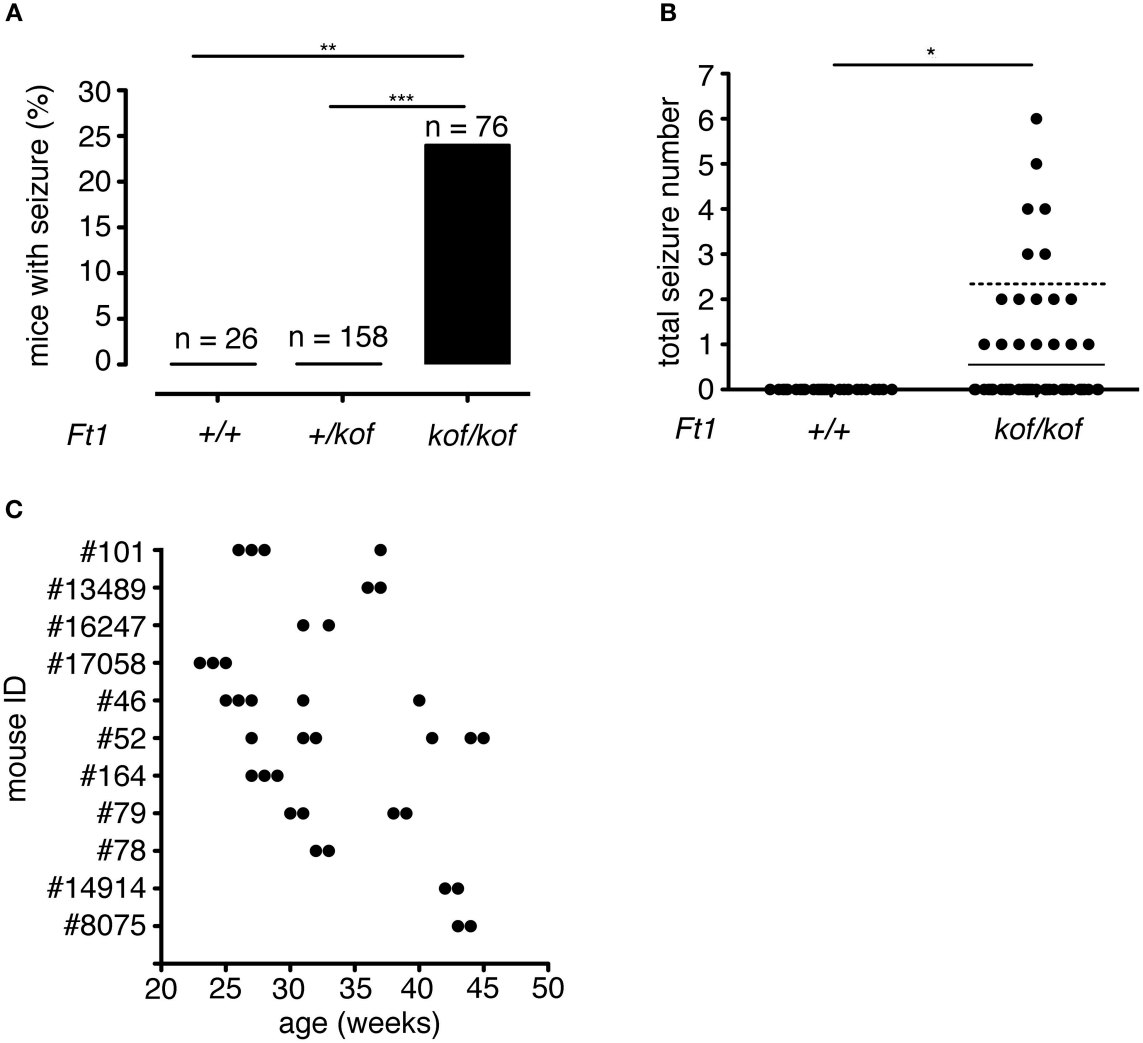
Seizure frequency in *Ft1*^*kof*/*kof*^ mice. **(A)** Percentages of mice that exhibited seizure; no seizures were observed in wt (**p < 0.01 χ^2^ test) or in heterozygous *Ft1*^+/*kof*^ mice (***p < 0.001 χ^2^ test). **(B)** Total number of seizure experienced by wt and *Ft1*^*kof*/*kof*^ mice, each dot represents one mouse. Line indicates average seizure number considering all *Ft1*^*kof*/*kof*^; dashed line indicates average seizure number (2.33 ± 0.14) considering *Ft1*^*kof*/*kof*^ which experienced seizures (*p < 0.05 Student's *t*-test). **(C)** Temporal seizure distribution for each *Ft1*^*kof*/*kof*^ mouse which experienced more than one seizure. Each dot represents a seizure episode. n, total number of analyzed mice.

Over a total of 260 animals (including *Ft1*^+/+^, *Ft1*^+/*kof*^, and *Ft1*^*kof*/*kof*^), 18 *Ft1*^*kof*/*kof*^ exhibited at least one seizure manifestation (Figures 2A,B). No seizure episodes were observed in heterozygote *Ft1*^+/*kof*^ or wt mice (Figure 2A). The frequency of seizures reached a maximum of one seizure episode per week. Of 18 seizure positive mutants, 7 exhibited one seizure-like manifestation during their follow-up period, while the leftover displayed more than one seizure. On average, we observed 2.33 ± 0.14 seizures during the entire follow-up period in the seizure positive mutants (Figure 2B). Time distribution of seizures for each mutant animal experiencing more than one seizure indicated non-random enrichment of seizures over the follow-up period, with 75% of seizures happening in consecutive weeks (Figure 2C).

### Seizures of Ft1 mutant mice are age and gender linked

**Figure 3:**
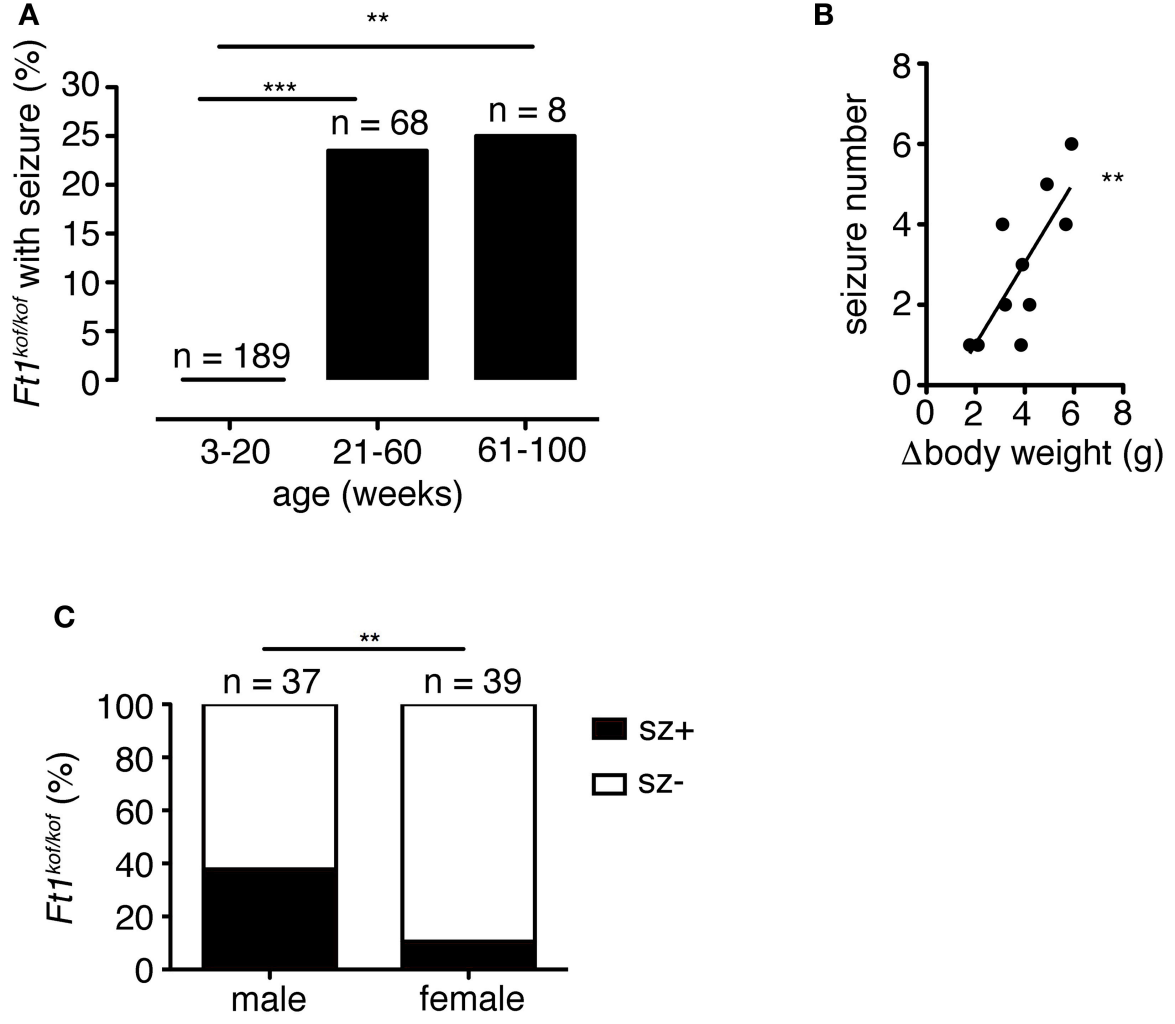
Seizures in *Ft1*^*kof*/*kof*^ mice are age, body weight and sex-linked. **(A)** Percentage of *Ft1*^*kof*/*kof*^ mice exhibiting first seizure clustered in three age intervals (young: 3 ≤ weeks ≤ 20; juvenile: 21 ≤ weeks ≤ 60; adult: 61 ≤ weeks ≤ 100). No seizures were observed in young animals (**p < 0.01 and ***p < 0.001 in χ^2^ test). **(B)** Correlation between the severity of growth defect and seizure frequency (r^2^ = 0.60; **p < 0.01 in Pearson's R test). Each dot represents data from an individual animal: body weight differences (Δbody weight) were obtained subtracting average body weight of *Ft1*^*kof*/*kof*^ to the average weight of age and sex-matched wt group. **(C)** Gender related differences in seizure behavior (**p < 0.01 in χ^2^ test). *n*, total number of analyzed mice.

To analyze the distribution of seizures during aging, we subdivided mouse lifespan in three major intervals: young (3 ≤ weeks ≤ 20), juvenile (21 ≤ weeks ≤ 60), and adult (61 ≤ weeks ≤ 100). We monitored, for each age interval, the fraction of mice exhibiting seizures for the first time (Figure 3A). None of young non SA *Ft1*^*kof*/*kof*^ mice (*n* = 189) displayed seizures, while seizures were observed in juvenile and adult mice, in 16 out of 68 and 2 out of 8, respectively.

We previously reported that non SA *Ft1*^*kof*/*kof*^ mice display growth defects, which start within the juvenile interval and become prominent through aging (La Torre et al., 2018). We then asked whether the seizure phenotype was paralleled by age-associated growth impairment. We considered the difference between the average body weight of wt (*n* = 6) and sex-matched seizure positive mutant mice (*n* = 8) (Δbody weight), and plotted it against the number of seizures observed in each mutant animal to generate a regression curve (Figure 3B). The two variables were linked by positive correlation (*r*^2^ = 0.60; ^**^p < 0.01 in Pearson's R test).

Non SA *Ft1*^*kof*/*kof*^ male mice were previously shown to display a stronger phenotype as compared to *Ft1*^*kof*/*kof*^ females (La Torre et al., 2018). We thus decided to explore gender differences in the seizure trait. We analyzed a cohort of non SA *Ft1*^*kof*/*kof*^ animals aged ≥ 21 weeks including 37 males and 39 females. Seizure positive mutants were 37.8% among males and 10.3% among non SA *Ft1*^*kof*/*kof*^ female mice (Figure 3C).

In order to verify whether aging would induce variations in Ft1 expression we monitored two cohorts of *Ft1*^+/+^ mice by QPCR. Results indicate that there are no significant variation in Ft1 expression with aging (Supplementary Figure 1A). In addition, the human counterpart of Ft1, AKTIP, is also expressed in brain cells (Supplementary Figure 1B).

Altogether these results indicate that *Ft1* reduction causes seizures, and that this phenotype parallels overall organismal alterations characteristic of non SA *Ft1*^*kof*/*kof*^ mice including age dependent growth impairment and sex-linked defects (La Torre et al., 2018).

### Structure of Ft1 mutant brain

**Figure 4:**
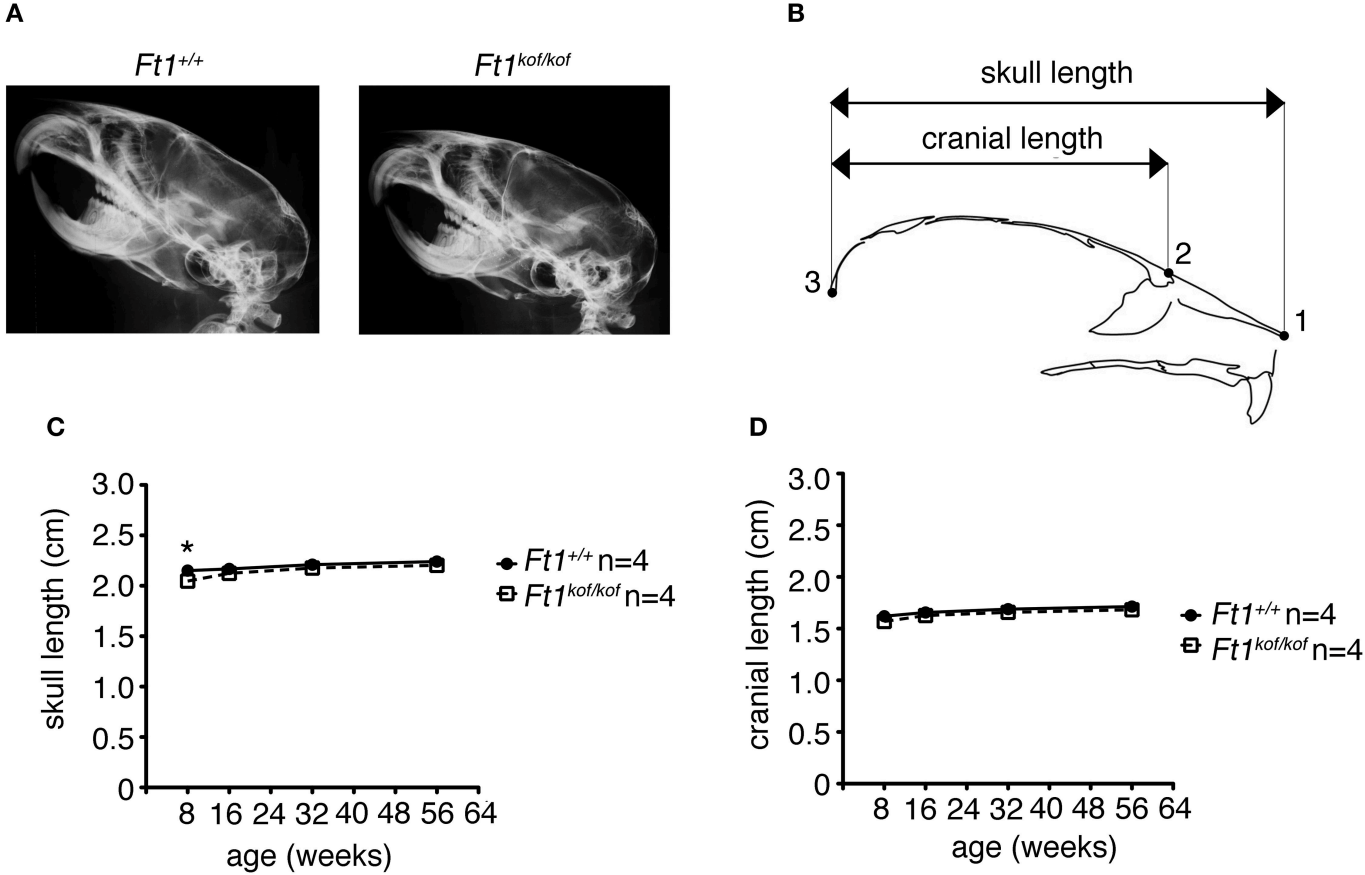
*Ft1* reduction does not affect skull development. **(A)** X-ray analysis of craniofacial skeleton of wt and *Ft1*^*kof*/*kof*^ mice. **(B)** Schematic view of mouse skull and description of landmarks used for the anterior-posterior craniofacial skeleton length analysis. **(C,D)** Skull and cranium length analysis of *Ft1*^*kof*/*kof*^ animals and age-matched wt controls. Curves are not overall significantly different in Student's *t*-test (*p* = 0.225), the difference of the skull length between *Ft1*^*kof*/*kof*^ and *Ft1*^+/+^ animals is significantly different at the age of 8 weeks (*p < 0.05 in Student's *t*-test). n, total number of analyzed mice.

**Figure 5:**
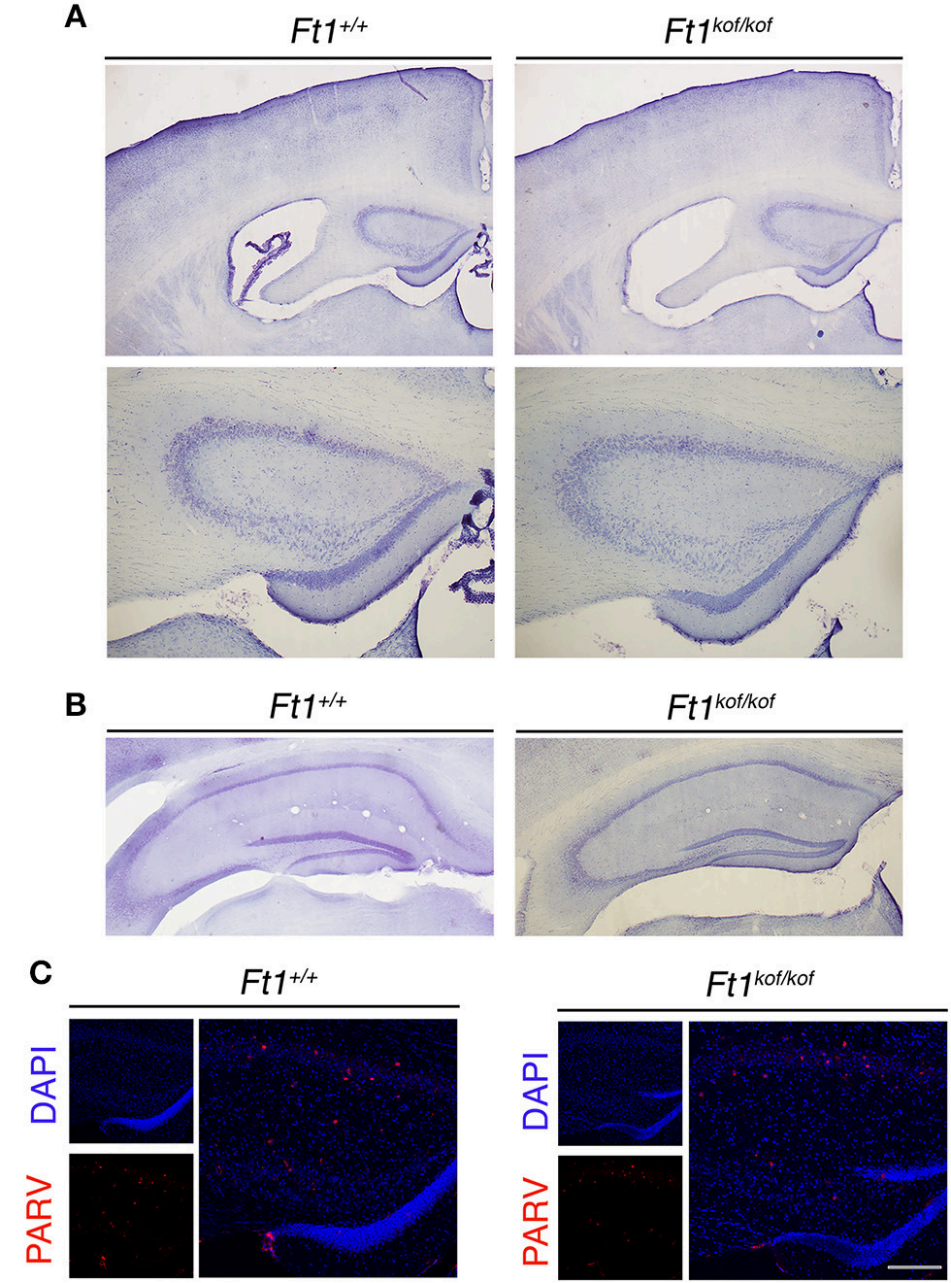
*Ft1* reduction does not overtly affect hippocampal organization. **(A)** Nissl-stained coronal sections of *Ft1*^+/+^ and *Ft1*^*kof*/*kof*^ mouse brains (upper panels), and higher magnification of the anterior hippocampus (bottom panels), at about Bregma −1.22 mm. **(B)** Nissl-stained hippocampus of *Ft1*^+/+^ and *Ft1*^*kof*/*kof*^ mice, at about Bregma −2.18 mm. **(C)** Confocal images showing parvalbumin-positive interneurons (PARV, in red) at the hippocampal level; cell nuclei are labeled by DAPI staining (in blue). Scale bars: **(A)** 500 μm (upper panel), **(A)** 200 μm (bottom panel), **(B)** 400 μm, **(C)** (merge) 160 μm, **(C)** (DAPI/PARV) 200 μm.

**Figure 6:**
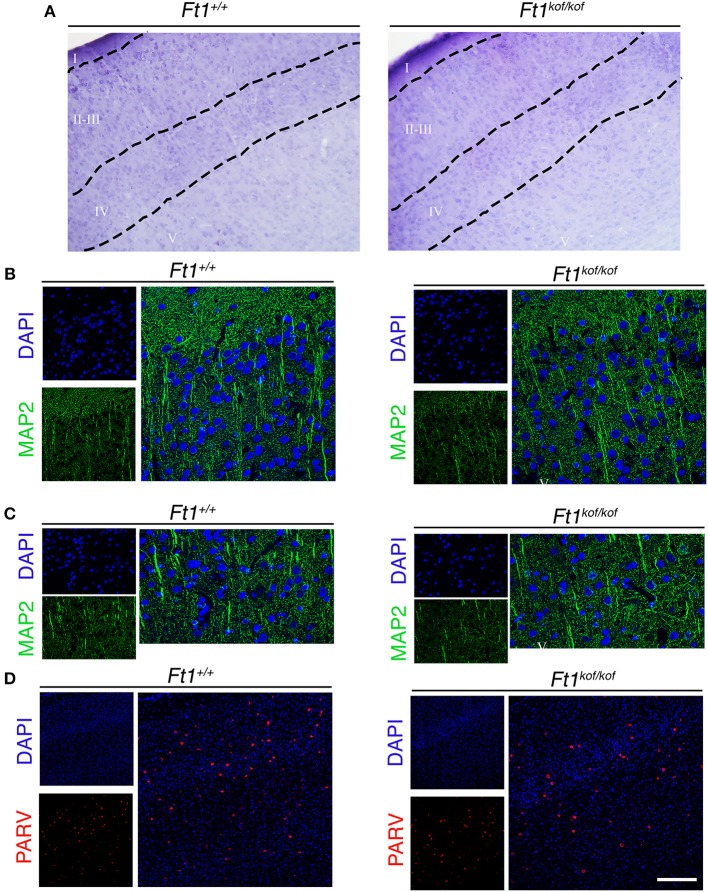
*Ft1* reduction does not affect somatosensory cortex cytoarchitecture. **(A)** Nissl-stained coronal sections, showing somatosensory cortex of *Ft1*^+/+^ and *Ft1*^*kof*/*kof*^ mice brains. **(B)** MAP2-positive neurons (green) located in the cortical supragranular layers I-II-III and **(C)** in the infragranular layer V; cell nuclei are labeled by DAPI staining (in blue). **(D)** Confocal images showing parvalbumin-positive interneurons (PARV, in red) and DAPI-positive nuclei (in blue). Scale bars: **(A)** 100 μm, **(B,C)** 55 μm, **(D)** 160 μm.

To define whether *Ft1* function impacted on brain structure we firstly measured skull and cranial length of non SA *Ft1*^*kof*/*kof*^ mice by X-ray analysis (Figure 4). Mutant and age- and sex-matched wt animals were monitored at 2, 4, 8, and 13 months. Length measures revealed a continuous increase during postnatal development in mutant mice as in controls, with a mild alteration induced by *Ft1* reduction at the age of 8 weeks (^*^p < 0.05 in Student's *t*-test) (Figures 4A–D). We then investigated brain morphology by histological analysis. We firstly evaluated the hippocampus, as hippocampal defects are often cause of epilepsy. Nissl-staining of coronal sections of *Ft1*^*kof*/*kof*^ brains did not highlight overt alterations of hippocampal organization or gross lesions, such as cell loss, structural deformation or scars (Figures 5A–B). By immunofluorescence, we further analyzed hippocampus cytoarchitecture. Semi-quantitative analysis of Parvalbumin-positive GABAergic interneurons showed a mild reduction in *Ft1*^*kof*/*kof*^ mice as compared to controls (Figure 5C and Supplementary Figure 2). Then we evaluated the somatosensory cortex cytoarchitecture: by Nissl-staining and MAP2 immunofluorescence, we did not detect evident alterations, including cortical thinning and layering defects in *Ft1*^*kof*/*kof*^ brain compared to wt (Figures 6A–C). The density of Parvalbumin-positive GABAergic interneurons in the cortex was non-significantly affected by *Ft1* reduction (Figure 6D and Supplementary Figure 3). Moreover, we did not observe any evident macroscopic alterations in the whole brain structure, as for example shrinkage of specific cerebral regions, ventricle enlargement, corpus callosum thinning (data not shown), or in brain volume (wt 177.95 mm^3^, *Ft1*^*kof*/*kof*^ 189.46 mm^3^; Supplementary Figure 4). Overall, these analyses did not reveal robust differences between non SA *Ft1*^*kof*/*kof*^ and control mice, thus in principle excluding the presence of severe brain degenerative processes.

### p53-sensitive inflammatory response in Ft1 mutant brain

**Figure 7:**
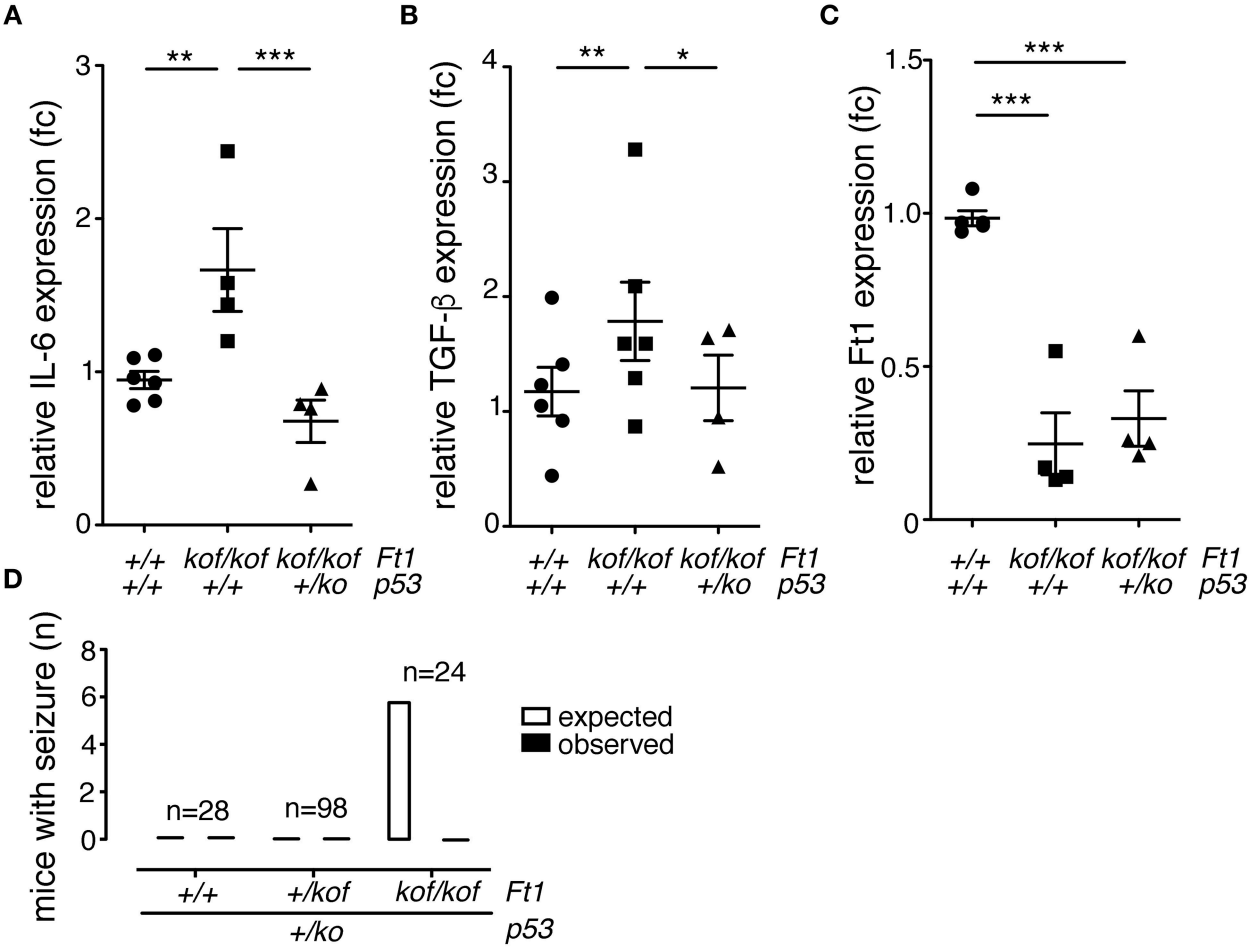
p53 reduction reverses inflammation and seizures in *Ft1*^*kof*/*kof*^ mice. **(A–C)** QPCR expression quantification of IL-6, TGF-β and *Ft1* in *Ft1*^*kof*/*kof*^; *Ft1*^+/*kof*^ *Ft1*^*kof*/*kof*^ in a wt or *p53*^+/*ko*^ background (*p < 0.05; **p < 0.01; ***p < 0.001 Student's *t*-test). **(D)** Expected versus observed number of mice with seizures in *Ft1*^*kof*/*kof*^; *Ft1*^+/*kof*^ *Ft1*^*kof*/*kof*^ in a wt or *p53*^+/*ko*^ background. Expected seizure number was calculated considering the seizure frequency in *Ft1*^*kof*/*kof*^ age matched cohort (see Figure 2A). To note that none of *Ft1*^*kof*/*kof*^ mice with the *p53*^+/*ko*^ mutation displayed seizures. n, total number of analyzed mice.

To investigate on molecular drivers for the seizure phenotype observed in *Ft1*^*kof*/*kof*^ mice we reasoned on studies in aging and progeroid models which link DNA damage to systemic inflammation and neurodegeneration (Campisi, 2013; López-Otín et al., 2013). Given the progeroid traits observed in non SA *Ft1*^*kof*/*kof*^ mice and the implication of *Ft1* in telomere metabolism and DNA function we decided to monitor the level of two canonical, interrelated, inflammatory cytokines: IL-6 and TGF-β. To get a full picture also on the connection with DNA damage, we explored this aspect in non SA *Ft1*^*kof*/*kof*^ mice, expressing normal levels of the guardian of the genome *p53* and in mice defective for *p53* expression due to heterozygous *p53* inactivation (*Ft1*^*kof*/*kof*^; *p53*^+/*ko*^). QPCR analysis showed significant higher levels of both IL-6 and TGF-β in *Ft1*^*kof*/*kof*^ brains as compared to age matched control mice (Figures 7A,B; ^**^p < 0.01). Indeed, in *Ft1*^*kof*/*kof*^; *p53*^+/*ko*^ mutant mice IL-6 and TGF-β activation was reversed. These results suggest that the activation of the DNA damage response pivotal player, *p53*, is a crucial event in the organismal response to *Ft1* reduction (Figures 7A,B). As expected, *p53* reduction did not impact on *Ft1* expression, which remained significantly reduced in *Ft1*^*kof*/*kof*^; *p53*^+/*ko*^ as in *Ft1*^*kof*/*kof*^ animals (Figure 7C).

Given the activation of the inflammatory cytokines and its rescue by p53 reduction, we decided to explore the link between p53, as an element directly associated with DNA function, to the seizure phenotype of *Ft1*^*kof*/*kof*^ mice. To this purpose, we analyzed three cohorts of ≥ 21 weeks mice including *Ft1*^+/+^;*p53*^+/*ko*^, *Ft1*^+/*kof*^;*p53*^+/*ko*^ and *Ft1*^*kof*/*kof*^;*p53*^+/*ko*^ mice, as compared to genetically and age matched controls. None of the mice with the *p53*^+/*ko*^ mutation displayed seizures, suggesting on one side that *p53* reduction alone does not cause seizures, and, on the other side, that it rescues the seizure phenotype of our *Ft1* mutant mice (Figure 7D).

Altogether these results establish a link among *Ft1, p53*, inflammatory parameters and seizure behavior.

## Discussion

Epilepsy is a complex disorder codified by the concept of predisposition to generate spontaneous seizures. Studies to interpret seizure etiology are complex in humans, indeed many open questions remain about its causes and mechanisms. An important tool to understand and interpret epileptic phenomena comes from model systems. Mutant mice in particular have been instrumental for the identification of genes and gene-determined molecular processes generating brain dysfunction and epileptic phenotypes (Fonck et al., 2005; Yu et al., 2006; Baraban, 2007; Löscher, 2011; Glasscock et al., 2012). In this work we report on the epileptic behavior of a mouse mutant obtained by genetically reducing the expression of the telomeric gene *Ft1* (AKTIP in humans) and present data supportive of a link between DNA damage and epileptic behavior.

As a consequence to its biological function, AKTIP/*Ft1* mutant cells display DNA replication defects, DNA and telomere damage, along with cell senescence (Burla et al., 2015, 2016a). AKTIP/*Ft1* is linked to nuclear architecture through the biochemical interaction occurring with A- and B-type lamins, the main components of the nucleoskeleton, which, in turn, is a pivotal element for the control of DNA function (Dittmer and Misteli, 2011; Burla et al., 2018). Mice with reduced *Ft1* expression display a segmental phenotype including reduced subcutaneous fat, growth and skeletal defects. These traits partly recapitulate the premature aging phenotypes observed in progeroid mice generated by mutations of lamins or DNA maintenance genes (Blasco, 2005; Stewart et al., 2007; Burla et al., 2018), and are exacerbated in adult individuals as compared to juvenile. Given the connection of AKTIP/*Ft1* with both lamins and DNA function, *Ft1* mutant mice represent an attractive model for investigating how these connections may impact on the organismal phenotype and on different tissues and organs.

We report here that *Ft1* mutant mice exhibit spontaneous seizures. Digital movies document the seizure manifestation highlighting typical traits, as loss of postural equilibrium, limb jerks, and convulsions, as in other epileptic mouse models (Yang et al., 2007; Minkeviciene et al., 2009; Chabrol et al., 2010; Glasscock et al., 2012; Simeone et al., 2018). Seizures were observed after the 20th week of age suggesting age-dependent degenerative changes. Seizures also correlated with growth impairment, which, in *Ft1*^*kof*/*kof*^ mice, is exacerbated with aging (La Torre et al., 2018). The frequency of seizures was higher in male mice, in line with the overall impact of *Ft1* reduction observed in our model (La Torre et al., 2018).

Along with behavioral descriptions, the importance of mouse models resides in the fact that they allow investigating upstream events to behavioral phenotypes, which, in humans, is complex to explore. We thus exploited these animals to analyze the pathophysiological path to seizures in an attempt to contribute to establish experimentally proven links between DNA function and brain alterations.

The analysis of brain morphology and cytoarchitecture in non SA epileptic *Ft1*^*kof*/*kof*^ mice did not highlight overt defects nor macroscopic neurodegeneration. These results were obtained by analyzing restricted brain areas and cell subtypes, and we do not exclude the possibility of other or more subtle brain alterations escaping our analysis. However, given the data, we hypothesized that molecular, rather than overt structural aberrations would underpin the epileptic behavior. This interpretation would be in line with the fact that many types of epilepsy have been associated with molecular alterations, rather than with macroscopic brain structure defects (Guerrini et al., 2014).

On the basis of this hypothesis, we investigated on putative molecular culprits for the seizure phenotype. Telomeres and cell senescence have been implicated in disease and aging. In particular, the secretion of a panel of inflammatory signals (Senescence Associated Secretory Phenotype, SASP) is considered a pivotal element in the alteration of tissue homeostasis contributing to pathological status (Campisi et al., 2001; Campisi, 2013). Given the function of AKTIP/*Ft1* in telomere protection along with its importance in preventing cell senescence and DNA damage (Burla et al., 2015; La Torre et al., 2018), we hypothesized that in *Ft1* mutant mice accumulation of damage and consequent senescence would provoke inflammatory cytokine production, which, in turn, could be implicated into the epileptic phenotype.

We thus measured inflammatory factors in the brain along with exploring the implication of DNA function in inflammation. In the brain of *Ft1* mutant mice we detected the activation of IL-6 and TGF-β. Both these factors are related with inflammation (Coppe et al., 2010). IL-6 activation is assigned to the SASP group of factors linked to cell senescence and has been defined as a senescence biomarker in mice (Coppe et al., 2010; Hudgins et al., 2018). The cytokine TGF-β, in association with IL-6, is a further inflammatory stimulus (Sanjabi et al., 2009). Altogether these data suggested a connection between seizures and inflammation in the brain of *Ft1*^*kof*/*kof*^ mice.

To further investigate on the causative cascade of pathologic events occurring in mice from *Ft1* mutation to seizures, we generated *Ft1* mutant mice with a heterozygous ko mutation in *p53*. p53 is a pivotal element in the DNA damage response and we have demonstrated that p53 is activated in AKTIP reduced primary human cells, which contributes to blocking cell proliferation and inducing senescence (Burla et al., 2015; La Torre et al., 2018). Interestingly *p53* loss was shown to rescue aging traits *in vivo*, by releasing DNA damage-induced checkpoints and cell senescence (Varela et al., 2005). In addition, p53 regulated senescence has been defined as a pivotal element in generating the SASP phenotype. Consistently, reduction of p53 expression rescues SASP related aging traits (Baar et al., 2017). In *Ft1* mutant mice, p53 reduction not only reversed back the expression of IL-6 and TGF-β, but also rescued the seizure phenotype. These results, taken together, induce to speculate that the path to seizures generated by Ft1 reduction could start with DNA damage, including telomere dysfunction, followed by p53 activation, cell senescence, and SASP. The latter could eventually induce brain seizures, through a mechanism that remains to be elucidated.

This interpretation of the data is interesting for investigating on seizure causative events in basal physiological conditions, but also in aging. In fact, aging is characterized by the exacerbation of the alteration of the biological pathways which we have taken into consideration, including senescence, genomic instability and telomere fragility (López-Otín et al., 2013). These factors act on aging in a cell intrinsic way and through extrinsic mechanisms (Lopez-Otin et al., 2016), as secretion of SASP factors (Coppe et al., 2010) and chronic inflammation, profoundly altering tissue microenvironment (Fulop et al., 2017), which, we would suggest, could impact also on brain function.

In conclusion, this work adds new insights into the connection among DNA damage, brain function, and inflammation. In addition, it further underscores the importance of model organisms for studying molecular path to specific phenotypes, along with permitting the study of genetic interactions at the organismal level. These aspects are even more relevant in aging and brain research studies for which work in humans is inaccessible due to time, ethical and sample accessibility issues.

## Materials and methods

### Mice and ethical statements

Mice were maintained and bred in a 12 h light/12 h dark cycle, in a pathogen free unit of the animal house at Biology and Biotechnology Department, Sapienza University. Animals were housed and treated in accordance with protocol 355/2017-PR approved by the Italian Ministry of Health. Animals carrying the knockout first mutations in the *Ft1* gene (*Ft1 kof*) were obtained as previously described (La Torre et al., 2018). In order to obtain *Ft1* and *p53* mutant animals *Ft1*^+/*kof*^ were crossed with *p53*^+/*ko*^ animals [kindly provided by G. Piaggio and S. Soddu IFO, Italy; (Jacks et al., 1994)]; subsequently doubly heterozygous mice were intercrossed to obtain the desired genotypes. Offspring were weaned at 3 weeks and tail biopsies were used for genotyping. When needed, mice were anesthetized by intramuscular Zoletil 20 (Virbac S.A., France), or euthanized by asphyxiation with carbon dioxide or cervical dislocation.

### Genotyping

Mice were genotyped as previously described (La Torre et al., 2018). Briefly genomic DNA was extracted from tail biopsies digested 50°C with a proteinase K/SDS solution using the blackPREP Rodent Tail DNA Kit (Analytik Jena, Jena, Germany) following manufacturer's instructions.

Mice were PCR genotyped using the following primers:

Ft1 E4 F: 5′-GTGAAGCAGAAGCTGCCAGGAGT−3′;
Ft1 E6 R: 5′-AGCTCACCCGAGGTGGGATCAA−3′;
p53-X6 F: 5′-AGCGTGGTGGTACCTTATGAGC−3′;
p53-Neo19 F: 5′-GCTATCAGGACATAGCGTTGGC−3′;
p53-X7 R: 5′-GGATGGTGGTATACTCAGAGCC−3′

### Seizure observation

Seizures were observed during routine mouse handling. Where indicated seizure and post-seizure events were video recorded. The movies were then analyzed for specific behavioral signs as previously described (Chabrol et al., 2010).

### X-ray and cephalometric measurements

X-ray images were taken using Faxitron MX-20 (Faxitron X-ray Corp.) at 24 kV for 6 s; images captured with Medical Imaging Film HM Plus (Ferrania). Skull length and cranial length were measured by Image J software as previously described (Rueden et al., 2017).

### Histological analysis

Eight month-old mice were euthanized and brains were removed and postfixed in 4% PFA for 2 h at 4°C. Samples were then immersed in a solution containing 30% sucrose in phosphate buffer 0.1 M at 4°C for cryoprotection, embedded in cryostat medium (Killik; Bio-Optica, Milan, Italy), and cut on the cryostat (HM 550; Microm) in serial transverse 50 μm thick sections. Some brain sections (one section every 600 μm) were Nissl-stained to evaluate the gross cerebral morphology: briefly sections were mounted on 2% gelatin-coated Superfrost slides and air-dried overnight; slides were hydrated in distilled water for 1 min before staining in 0.1% Cresyl violet acetate (Sigma Aldrich, St. Louis, MO) for 10 min, dehydrated in an ascending series of ethanol, cleared in xylene and cover-slipped with Eukitt (Bioptica, Milan, Italy). Sections were examined at a Nikon Eclipse 80i microscope Equipped with a Nikon DS-Fi1 camera. Brain volume of wt and *Ft1*^*kof*/*kof*^ mice was calculated considering Bregma 2.10 mm to Bregma −2.54 mm segments in Nissl-stained serial sections reconstructed by Neurolucida software (MicroBrightField, Williston, VT, USA) and the volume (expressed in mm^3^) was obtained by NeuroExplorer software (MicroBrightField). For immunofluorescence, brain sections were permeabilized with in PBS 0.3% Triton X-100 at RT on a tilting plate for 20 min; then, to block unspecific binding of the antibody, sections were incubated for 30 min at RT with 0.3% Triton X-100 and 10% normal donkey serum or normal goat serum (Sigma-Aldrich) in PBS (pH 7.4). Sections were then incubated at 4°C overnight with the following antibodies: 1:7,500 anti-Parvalbumin (rabbit; Swant); 1:200 anti-MAP2 (mouse; Chemicon). Then, sections were incubated with appropriate fluorochrome-conjugated secondary antibodies, for 1h at RT: 1:400 Alexa Fluor 647, goat anti mouse (Jackson ImmunoResearch Laboratories); 1:200 cyanine 3-conjugated secondary antibody, donkey anti-rabbit (Jackson ImmunoResearch Laboratories); 1:200 cyanine 2-conjugated secondary antibody, donkey anti-mouse (Jackson ImmunoResearch Laboratories). Finally sections were incubated with 4,6 Diamino-2 phenyindole Dilactate (DAPI; Sigma Aldrich) in PBS 1:50 for 3 min. Samples were washed and coverslips were mounted with the anti-fade mounting medium Mowiol. For double staining and 3D reconstructions, slides were examined with a Leica TCS SP5 confocal laser scanning microscope.

### Cell culture

Human foreskin primary fibroblasts (HPFs), HeLa (ATCC CCL-2) and 293T (ATCC CRL-11268) cells were cultured in DMEM with 10% FBS. SH-SY5Y (ATCC CRL-2266) cells were cultured in EMEM supplemented with 10% FBS.

### RNA extraction and QPCR analysis

Cells were lysed using the TRIzol reagent (Invitrogen). Brains were removed from euthanized mice and frozen in liquid nitrogen. RNA was extracted using the TRIzol reagent (Invitrogen) according to manufacturer. After DNaseI treatment (Invitrogen) RNA from cells and brains was reverse transcribed into cDNA with oligo d(T) primer and OMNISCRIPT RT KIT (Qiagen). QPCRs were performed as described (Piersanti et al., 2015) using the following primers:

Ft1 E3 F: AACCAGTCCTCCACGAAGTGCA;
Ft1 E3 R: TAGGGCTTCGCTATGGGTAGAGCA;
Ft1 E6 F: CCGTCTTTCACCCACTAGTTGAT;
Ft1 E6 R: TTGCGAACGCTCTTTTCACA;
mGAPDH F: GTGGCAAAGTGGAGATTGTTGCC;
mGAPDH R: TGTGCCGTTGAATTTGCCGT;
IL-6 F: CTCTGGGAAATCGTGGAAAT;
IL-6 R: CCAGTTTGGTAGCATCCATC
TGF-β F: CCCTATATTTGGAGCCTGGA;
TGF-β R: CTTGCGACCCACGTAGTAGA;
AKTIP F: TCCACGCTTGGTGTTCGAT;
AKTIP R: TCACCTGAGGTGGGATCAACT
GAPDH F: TGGGCTACACTGAGCACCAG
GAPDH R: GGGTGTCGCTGTTGAAGTCA

### Statistics

χ^2^ test was applied for comparisons of the mouse cohorts. Independent data sets were analyzed with the Student's *t*-test (unpaired, two-tailed). Correlation analyses were performed via Pearson's R test.

## Ethics statement

This study was carried out in accordance with the recommendations of the European Directive 2010/63/EU on the protection of animals used for scientific purposes. The protocol was approved by the Italian Ministry of Health (Ministero della Salute) with protocol number 355/2017-PR.

## Author contributions

RB, MLT, FDC, AV, and FV contributed to the design of the experiments and to the writing of the manuscript. MLT, GZ, AB, SDG, MB, and CM performed the experiments and analyzed the data. IS designed the experiments, analyzed the data and wrote the manuscript.

### Conflict of interest statement

The authors declare that the research was conducted in the absence of any commercial or financial relationships that could be construed as a potential conflict of interest.
