# Supplementary material for: p53-Sensitive Epileptic Behavior and Inflammation in Ft1 Hypomorphic Mice
Source: Front Genet. 2018 Nov 27;9:581. doi: 10.3389/fgene.2018.00581 (PMC6278696; doi:10.3389/fgene.2018.00581)
Supplement: Supplementary file 1 [file Table_1.docx]

Supplementary Material

**p53-sensitive epileptic behavior and inflammation**

**in *Ft1* hypomorphic mice**

**Romina Burla^1#^, Mattia La Torre^1#^, Giorgia Zanetti^1^, Alex Bastianelli^1^, Chiara Merigliano^1,3^, Simona del Giudice^1^, Alessandro Vercelli^2^, Ferdinando Di Cunto^2^, Marina Boido^2^, Fiammetta Vernì^1^ and Isabella Saggio^1,3,*^**

^1^ Department of Biology and Biotechnology Sapienza University of Rome, Italy

^2^ Neuroscience Institute Cavalieri Ottolenghi, Orbassano (Torino) and Department of Neuroscience, University of Torino, Italy

^3^ Nanyang Technological University, Singapore

#co-first authors

***Correspondence:**

[isabella.saggio@uniroma1.it](mailto:isabella.saggio@uniroma1.it)

# Keywords: aging, epilepsy, DNA damage, p53, DNA repair

# Supplementary Data

**Supplementary movies (relative to Figure 1): Seizure and post-seizure video recording of *Ft1^kof/kof^* mice** Supplementary movies 1-3: seizure videos recording of *Ft1^kof/kof^* mice (movies 1-2 mouse ID #13489, movie 3 mouse ID #16247). Supplementary movies 4-6: post-seizure videos recording *Ft1^kof/kof^* mice (movies 4-5 mouse ID #13489, movie 6 mouse ID #16247).

# Supplementary Figures


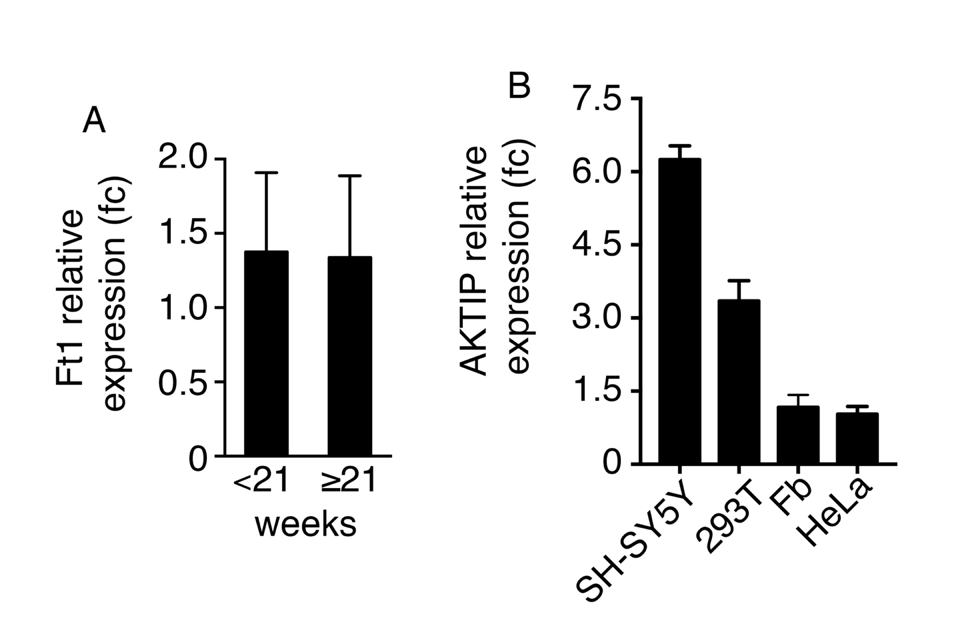


**Supplementary Figure 1 QPCR analysis of AKTIP/Ft1 expression in mice and human cells** (A) Ft1 expression in wt mice is not significantly different between < 21 weeks and ≥ 21 weeks old mice. (B) AKTIP expression in different human cell types: SH-SY5Y, 293T, HeLa cell lines and primary fibroblast (Fb). AKTIP expression in Fb, in which a full AKTIP dependent DNA damage phenotype was described (Burla et al., 2015), was considered as the reference value for the analysis. The differences in AKTIP expression between analyzed cell types were statistically significant with a p value <0.05 (Student’s t-test) except for the difference between Fb and HeLa cell line.


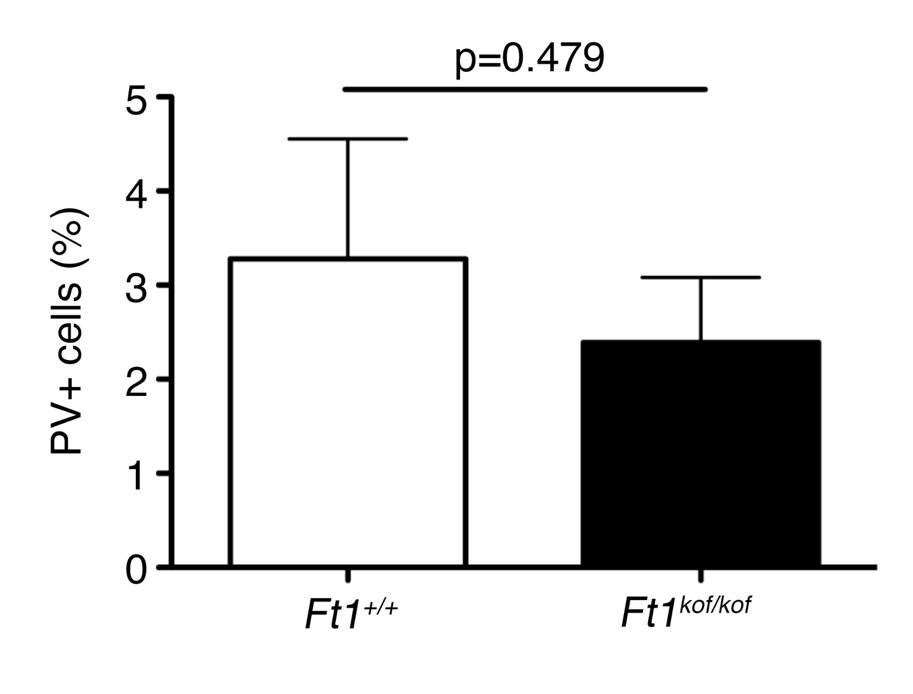


**Supplementary Figure 2** **(relative to Figure 5) Semi-quantitative histological analysis of hippocampal sections of *Ft1^kof/kof^* and wt brains** Percentage of Parvalbumin-positive GABAergic interneurons (PV+ cells) residing in the CA1 hippocampal subfield, indicated as the number of PV+ cells divided for the total number of the cells (4 sections, p=0.479 in Student’s t-test).

**
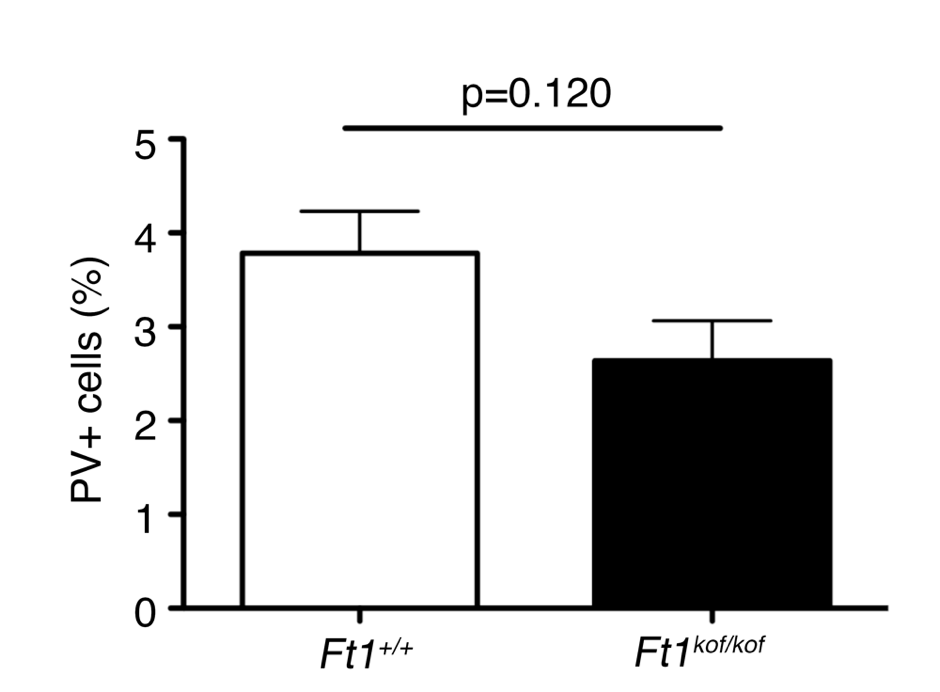
**

**Supplementary Figure 3 (relative to Figure 6)** **Semi-quantitative histological analysis of the somatosensory cortex of *Ft1^kof/kof^* and wt brains** Percentage of Parvalbumin-positive GABAergic interneurons (PV+ cells) residing in the somatosensory cortex, indicated as the number of PV+ cells divided for the total number of the cells (4 sections, p=0.120 in Student’s t-test).


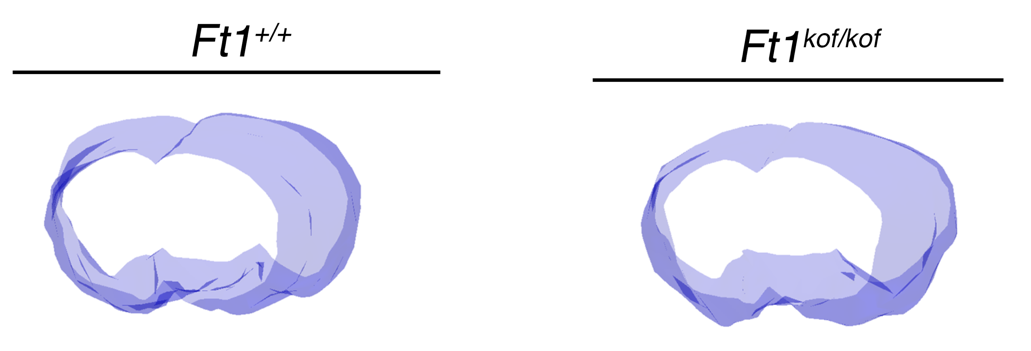


**Supplementary Figure 4 Brain volume analysis of *Ft1^kof/kof^* and wt mice** 3D reconstructions of wt and *Ft1^kof/kof^* brains (segment analyzed: from Bregma 2.10 mm to Bregma -2.54 mm). Brain volumes were calculated considering Bregma 2.10 mm to Bregma -2.54 mm segments in Nissl-stained serial sections reconstructed by Neurolucida software (MicroBrightField, Williston, VT, USA) and the volume (expressed in mm^3^) was obtained by NeuroExplorer software (MicroBrightField).
